# The increased risk of multiple sclerosis associated with HLA-DRB1*15:01 and smoking is modified by alcohol consumption

**DOI:** 10.1038/s41598-021-00578-y

**Published:** 2021-10-27

**Authors:** Anna Karin Hedström, Tomas Olsson, Lars Alfredsson

**Affiliations:** 1 Department of Clinical Neuroscience, K8, Karolinska Institutet, 171 77 Stockholm, Sweden; 2 Neuroimmunology Unit, Department of Clinical Neuroscience and Center for Molecular Medicine, Karolinska Institutet at Karolinska University Hospital, Solna, Sweden; 3 Department of Clinical Neuroscience and Institute of Environmental Medicine, Karolinska Institutet, Stockholm, Sweden

**Keywords:** Neurology, Risk factors

## Abstract

Previous studies have observed an inverse association between alcohol consumption and multiple sclerosis (MS) risk. We aimed to investigate possible interactions between alcohol consumption, MS-associated human leukocyte antigen (HLA) genes and smoking regarding MS risk. We used a Swedish population-based case–control study (2059 incident cases, 2887 controls) matched by age, sex, and residential area. Subjects with different genotypes and alcohol consumption habits were compared regarding MS risk, by calculating odds ratios with 95% confidence intervals using logistic regression models. Interaction on the additive scale between non-drinking and both genotype and smoking were assessed by calculating the attributable proportion due to interaction (AP). There was a dose-dependent inverse association between alcohol consumption and MS risk (*p* for trend < 0.0001). A potentiating effect was observed between non-drinking and presence of DRB1*15:01 (AP 0.3, 95% CI 0.2–0.5) which was of similar magnitude irrespective of smoking habits. Non-drinking also interacted with smoking to increase MS risk (AP 0.2, 95% CI 0.06–0.4). Non-drinking interacts with DRB1*15:01 and smoking to increase the risk of MS. Better understanding of the mechanisms behind our findings may help to define ways to achieve protection against MS by other means than alcohol consumption.

## Introduction

The etiology of multiple sclerosis (MS) involves both environmental and genetic factors, and interactions between them. The strongest genetic associations with MS are located within the human leukocyte antigen (HLA) complex. The primary risk allele DRB1*15:01 is located within the HLA class II region and increases the risk of disease development by threefold, whereas the most significant protective allele A*02:01 is located within the class I region^1,2^.

The MS-associated HLA genes DRB1*15:01 and absence of A*02:01 have been demonstrated to interact with several environmental factors in MS development, including high EBNA-1 antibody levels^3–5^, infectious mononucleosis^5,6^, adolescent obesity^7^, smoking^8,9^, exposure to passive smoking^10^ and organic solvents^11^.

Alcohol exposure dose-dependently affects all components of the adaptive immune system^12–14^. However, the impact of alcohol consumption on MS risk remains poorly understood. Results from small studies that have previously investigated the relationship between alcohol intake and MS risk have been inconsistent^15,16^. Alcohol consumption was not related to MS risk in a prospective cohort study^17^. In our Swedish case–control studies EIMS (Epidemiological Investigation of Multiple Sclerosis) and GEMS (Genes and Environment in Multiple Sclerosis), alcohol consumption was associated with reduced risk of MS^18^, which is in line with a recent Danish case–control study in which alcohol in adolescence was associated with lower risk of subsequent MS^19^.

In the present study, we used an updated version of the ongoing EIMS in order to study potential interactions between non-drinking and both MS-associated HLA genes and smoking with regard to MS risk.

## Methods

### Design and study population

EIMS is a population-based case–control study on environmental and genetic risk factors for MS. The study group comprises the Swedish population aged 16–70 years. Incident cases of MS were recruited via 42 hospital-based neurology units, including all university hospitals in Sweden. All cases were examined and diagnosed by a neurologist located at the unit where the case was recruited. Only cases who fulfilled the McDonald criteria were included in the present report. For each case, two controls were randomly selected from the national population register, matched by age in 5-year age strata, sex and residential area. The study period was April 2005 to June 2015. The EIMS project has been described in detail elsewhere^6–11^. Ethical approval was obtained by the Regional Ethical Review Board at Karolinska Institutet (2004–252/1–4). All participants provided their informed consent to participate after the purpose of the study was explained. According to the Swedish Ethical Review Authority, persons 15–18 years can provide informed consent (2003:460, paragraph 18). The paragraph refers to The Act concerning the Ethical Review of Research Involving Humans. All methods were carried out in accordance with relevant guidelines and regulations.

### Data collection

Information regarding demographic factors, environmental exposures, and lifestyle habits was collected using a standardized questionnaire. The questionnaires were filled out at the time of diagnosis, with a median time of 1.0 year between the onset of MS symptoms and the diagnosis. Subjects were asked to provide information regarding current alcohol consumption and whether their current alcohol consumption differed from their alcohol consumption five years ago. Completed questionnaires were obtained from 2880 cases and 6122 controls, the response proportion being 93% for the cases and 73% for the controls.

### Genotyping and measurement of EBNA-1 antibody levels

Blood samples were available for 2059 cases and 2887 controls. HLA-DRB1 and HLA-A alleles were determined at four-digit resolution. Genotyping was performed on the MS replication chip^20^ which is based on an Illumina exome chip to which approximately 90,000 custom markers were added and HLA was then imputed with HLA*IMP:02^21^. The reference panel was enriched with 400 Swedish controls for MHC class II alleles derived from sequence of exome 2 in order to improve imputation quality^22^. Antibodies against the primary EBNA-1 peptide segment associated with MS risk (aa 385–420)^23^ was detected using a multiplex serological assay, which has been described in more detail elsewhere^24^. Dual-laser flow-based detection was used to quantify the antibodies as units of median flourescence intensity.

### Definition of alcohol consumption and smoking habits

Based on current alcohol consumption habits, those who drank alcohol were defined as drinkers and those who did not drank alcohol were defined as non-drinkers. We further categorized the drinkers into the following subgroups based on the amount of alcohol intake per week: low consumption (< 50 g/wk for women and < 100 g/wk for men), moderate consumption (50–112 g/wk for women and 100–168 g/wk for men), and high consumption (> 112 g/wk for women and > 168 g/wk for men). The cutoffs were the same as those used by Statistics Sweden.

For each case, the year of disease onset was defined as the index year and the corresponding controls were given the same index year. Smoking was only considered before the index year. Those who were smoking at the time of the index year were defined as current smokers, those who had stopped smoking before the index year were defined as past smokers and those who had never smoked before or at the index year were defined as never smokers.

### Statistical analysis

Subjects with different genotypes and alcohol consumption habits were compared with regard to MS risk, by calculating odds ratios (OR) with 95% confidence intervals (CI) using unconditional logistic regression models^25^. Trend test for a dose response relationship regarding weekly amount of alcohol intake and MS risk was performed by using a continuous variable for alcohol consumption (gram/week) in a logistic regression model.

Potential interactions on the additive scale, defined by departure from additivity of effects, between genotype and alcohol, and between smoking and alcohol, were assessed by calculating the attributable proportion due to interaction (AP) with 95% CI. The AP between two interacting factors reflects the joint effect beyond the sum of the independent effects.

All analyses were adjusted for age, sex, residential area, and ancestry, and when appropriate for smoking and DRB1*15:01. Age was categorized into the following 8 strata: < 20, 20–24, 25–29, 30–34, 35–39, 40–45, 45–49, and 50 + years of age. Ancestry was dichotomized into Swedish and non-Swedish origin. A subject who was born in Sweden, whose parents had not immigrated, was classified as Swedish. Adjustments were also made for current civil status (single or living with an adult), heredity (having a first or second degree relative with MS or not), educational level (having a university degree or not), socioeconomic index, snuff use (yes or no), adolescent body mass index, EBNA-1 antibody levels, and sun exposure habits. The last occupation during the year before the index year was used as a marker for socioeconomic class which was categorized into the following strata: 1, workers in goods production; 2, workers in service production; 3, employees at lower and intermediate levels; 4, employees at higher levels, executives, university graduates, and 5; others such as pensioners, students, and unemployed. Adolescent body mass index was calculated by dividing self-reported weight in kilograms at age 20 years by self-reported height in meters squared and categorized into underweight (< 18.5 kg/m^2^), normal weight (18.5–25 kg/m^2^), overweight (25–30 kg/m^2^) and obese (> 30 kg/m^2^). EBNA-1 antibody levels were dichotomized into high or low, based on the median flourescence intensity among controls. Based on three questions regarding sun exposure during the last five years, where each answer alternative was given a number ranging from 1 (the lowest exposure) to 4 (the highest exposure), we constructed an index by adding the numbers together and thus acquired a value between 3 and 2. Sun exposure was then dichotomized into high or low exposure (index value more or less than 6). However, these factors had only minor influence on the results and were not kept in the final model.

The analyses on alcohol consumption and MS risk were also performed restricted to subjects with an index year within the previous five years who reported no change in alcohol habits during this period.

The main analyses were also performed comparing high and low consumers of alcohol. We thus studied the potential interaction between low alcohol consumption and DRB1*15:01 using DRB1*15:01 negative subjects with high alcohol consumption as the reference group. Similarly, we studied the potential interaction between low alcohol consumption and smoking, with high consumers of alcohol who did not smoke as the reference group. All analyses were conducted using Statistical Analysis System (SAS) version 9.4.

### Ethics approval

Ethical approval was obtained by the Regional Ethical Review Board at Karolinska Institutet.

### Consent to participate

All participants provided written consent.

## Results

Our analyses on alcohol consumption and MS risk included 2059 cases and 2887 controls. The mean age at onset was 34.4 years and the median duration from disease onset to the diagnosis was 1.0 year. Characteristics of cases and controls, in total and by alcohol consumption habits, are presented in Table 1. Both past and current smoking was more common among drinkers than among non-drinkers.

**Table 1:** Characteristics for cases and controls, by alcohol consumption habits.

Variable	Total		Non-drinkers		Drinkers	
	Cases	Controls	Cases	Controls	Cases	Controls
N	2059	2887	627	784	1432	2103
Women, n (%)	1491 (72)	2166 (75)	493 (79)	657 (84)	998 (70)	1509 (72)
Men, n (%)	568 (28)	721 (25)	134 (21)	127 (16)	434 (30)	594 (28)
Swedish, n (%)	1625 (79)	2209 (77)	465 (74)	553 (71)	1160 (81)	1656 (79)
Ever smoking, n (%)	1093 (53)	1286 (45)	291 (46)	259 (33)	802 (56)	1027 (49)
Past smoking, n (%)	441 (21)	602 (21)	118 (19)	122 (16)	323 (23)	480 (23)
Current smoking, n (%)	652 (32)	684 (24)	173 (28)	137 (17)	479 (34)	547 (26)
BMI at age 20 years, kg/m^2^ (SD)	22.6 (3.9)	21.8 (3.1)	23.0 (4.6)	21.9 (3.6)	22.4 (3.6)	21.7 (3.0)
DRB1*1501, n (%)	1123 (55)	802 (28)	347 (55)	190 (24)	776 (54)	612 (29)
A*0201, n (%)	852 (41)	1563 (54)	253 (40)	403 (51)	599 (42)	1160 (55)
Age at onset (SD)	34 (11)		33 (11)		35 (11)	

There was a dose-dependent inverse association between alcohol consumption and MS risk (*p* for trend < 0.001). Overall, alcohol consumption was associated with a 20% reduced risk of MS (OR 0.8, 95% CI 0.7–0.9) compared with never-drinkers. The risk reduction was more pronounced among ever smokers (OR 0.7, 95% CI 0.6–0.8) than among never smokers (OR 0.9, 95% CI 0.7–1.0).

A potentiating effect was observed between non-drinking and presence of DRB1*15:01 (AP 0.3, 95% CI 0.2–0.5) which was of similar magnitude irrespective of smoking habits (Table 2). No interaction was found between non-drinking and absence of A*02:01 (AP 0.08, 95% CI -0.1–0.3) (eTable 1).

**Table 2:** OR with 95% CI of developing MS for subjects with different DRB1*15:01 status and alcohol consumption habits.

DRB1*15:01	Alcohol	ca/co^a^	OR (95% CI)^b^	AP (95% CI)
−	+	656/1491	1.0 (reference)	
−	−	280/594	1.2 (1.0–1.4)	
+	+	776/612	2.9 (2.5–3.4)	
+	−	347/190	4.4 (3.6–5.4)	0.3 (0.2–0.5)

DRB1*15:01	Alcohol	ca/co^a^	OR (95% CI)^c^	AP (95% CI)
**Never smokers**				
−	+	276/757	1.0 (reference)	
−	−	150/402	1.0 (0.8–1.3)	
+	+	354/319	3.1 (2.5–3.8)	
+	−	186/123	4.2 (3.2–5.4)	0.3 (0.04–0.5)

DRB1*15:01	Alcohol	ca/co^a^	OR (95% CI)^c^	AP (95% CI)
**Ever smokers**				
−	+	380/734	1.0 (reference)	
−	−	130/192	1.3 (1.0–1.7)	
+	+	422/293	2.8 (2.3–3.5)	
+	−	161/67	4.8 (3.5–6.6)	0.3 (0.1–0.6)

^a^number of exposed cases and controls; ^b^adjusted for age, sex, residential area, ancestry, and smoking; ^c^adjusted for age, sex, residential area, and ancestry; AP = attributable proportion due to interaction.

Attributable proportion due to interaction between presence of DRB1*15:01 and non-drinking.

Overall, non-drinking interacted with smoking to increase MS risk (AP 0.2, 95% CI 0.06–0.4). The interaction seemed to occur independently of DRB1*15:01 status (Table 3). The interaction between non-drinking and smoking with regard to MS risk was somewhat more pronounced among current smokers (AP 0.3, 95% CI 0.05–0.5) than among past smokers (AP 0.2, 95% CI 0.002–0.5) (Table 4). The interaction between non-drinking and smoking also became significantly stronger with cumulative dose of smoking (eTable 2).

**Table 3:** OR with 95% CI of developing MS for subjects with different smoking and alcohol consumption habits, by DRB1*15:01 status.

Ever smoking	Alcohol	ca/co^a^	OR (95% CI)^b^	AP (95% CI)
−	+	630/1076	1.0 (reference)	
−	−	336/525	1.2 (1.0–1.4)	
+	+	802/1027	1.4 (1.2–1.6)	
+	−	291/259	2.1 (1.7–2.5)	0.2 (0.06–0.4)

Ever smoking	Alcohol	ca/co^a^	OR (95% CI)^c^	AP (95% CI)
DRB1*15:01 negative				
−	+	276/757	1.0 (reference)	
−	−	150/402	1.1 (0.8–1.4)	
+	+	380/734	1.4 (1.2–1.7)	
+	−	130/192	2.0 (1.5–2.6)	0.2 (0.003–0.6)

Ever smoking	Alcohol	ca/co^a^	OR (95% CI)^c^	AP (95% CI)
DRB1*15:01 positive				
−	+	354/319	1.0 (reference)	
−	−	186/123	1.3 (1.0–1.8)	
+	+	422/293	1.4 (1.1–1.7)	
+	−	161/67	2.3 (1.6–3.1)	0.2 (− 0.03–0.5)

^a^number of exposed cases and controls; ^b^adjusted for age, sex, residential area, ancestry, and HLA-DRB1*15:01; ^c^adjusted for age, sex, residential area, and ancestry; AP = attributable proportion due to interaction.

Attributable proportion due to interaction between smoking and non-drinking.

**Table 4:** OR with 95% CI of developing MS for subjects with different smoking and alcohol consumption habits.

	Alcohol	ca/co^a^	OR (95% CI)^b^	AP (95% CI)
**Ever smoking**				
−	+	630/1076	1.0 (reference)	
−	−	336/525	1.2 (1.0–1.4)	
+	+	802/1027	1.4 (1.2–1.6)	
+	−	291/259	2.1 (1.7–2.5)	0.2 (0.06–0.4)
**Past smoking**				
−	+	630/1076	1.0 (reference)	
−	−	336/525	1.2 (1.0–1.4)	
+	+	323/480	1.3 (1.1–1.5)	
+	−	118/122	1.8 (1.4–2.4)	0.2 (0.002–0.5)
**Current smoking**				
−	+	630/1076	1.0 (reference)	
−	−	336/525	1.2 (1.0–1.4)	
+	+	479/547	1.5 (1.3–1.8)	
+	−	173/137	2.3 (1.8–3.0)	0.3 (0.05–0.5)

^a^number of exposed cases and controls; ^b^adjusted for age, sex, residential area, ancestry, and HLA-DRB1*15:01; AP = attributable proportion due to interaction.

Attributable proportion due to interaction between smoking and non-drinking.

When different categories of DRB1*15:01 status, smoking and alcohol consumption were compared, DRB1*15:01 positive smokers who reported non-drinking had a sevenfold increased risk of developing MS (OR 7.1, 95% CI 5,1–9,8), compared to DRB1*15:01 negative never-smokers who reported drinking (Table 5).

**Table 5:** OR with 95% CI of developing MS for subjects with different combinations of DRB1*15:01 status, smoking and alcohol consumption habits.

DRB1*15:01	Smoking	Alcohol	ca/co^a^	OR (95% CI)^b^
−	−	+	276/757	1.0 (reference)
−	+	+	380/734	1.5 (1.2–1.8)
+	−	+	354/319	3.0 (2.5–3.7)
+	+	+	422/293	4.2 (3.4–5.2)
−	−	−	150/402	1.0 (0.8–1.3)
−	+	−	130/192	1.9 (1.5–2.5)
+	−	−	186/123	4.3 (3.3–5.6)
+	+	−	161/67	7.1 (5.1–9.8)

^a^number of exposed cases and controls; ^b^adjusted for age, sex, residential area, and ancestry.

Our findings remained similar when the analyses were restricted to include subjects with an index year within five years prior to inclusion in the study, who reported that they had not changed their alcohol consumption habits during the past five years (eTable 3).

When the analyses were restricted to include high and low alcohol consumers, an interaction was observed between low alcohol consumption and presence of DRB1*15:01 compared to the reference group of DRB1*15:01 negative low consumers of alcohol (AP 0.3, 95% CI 0.009–0.5). Similarly, an interaction was present between low alcohol consumption and smoking (AP 0.3, 95% CI 0.03–0.5) (eTables 4 and 5).

## Discussion

Low and moderate alcohol consumption has been shown to attenuate innate inflammatory responses in humans^12–14^ which is compatible with our finding that alcohol consumption is inversely related to MS risk. We also observed interactions between presence of DRB1*15:01 and non-drinking as well as between smoking and non-drinking regarding MS risk.

The interaction between non-drinking and smoking was less pronounced among past smokers than among current smokers, although still significant. This finding may not be surprising considering the long-lasting detrimental effect of smoking on MS risk after smoking cessation^26^.

Alcohol and its metabolite acetate have been demonstrated to reduce the production of interleukin-21, a major immune modulator that may enhance autoimmune responses through various mechanisms, such as development and activation of helper T-17 and follicular helper T cells and suppression of regulatory T cells^27^. Interleukin-21 has been suggested to be involved in the induction of several autoimmune diseases and has also been correlated with both MS severity and progression^28,29^.

With regard to risk of developing rheumatoid arthritis, a similar interaction has been reported between DRB1 SE genes and non-drinking, and among DRB1 SE positive subjects, an interaction was also found between smoking and non-drinking^30^. Alcohol thus seems to have a similar impact on both MS and rheumatoid arthritis, which are both complex Th1 driven inflammatory diseases. Knowledge concerning the molecular mechanisms underlying the specific interactions presented in this report is limited but may involve epigenetic mechanisms^31,32^.

All studies that have been conducted regarding the influence of alcohol consumption on MS disease course have shown that alcohol consumption is associated with reduced progression of disability^33–37^. Together with our findings, these data are relevant for clinical practice and give no support for advising healthy individuals with MS heredity, or individuals with MS, to completely refrain from alcohol. However, consumption of alcohol has detrimental effects on other disease conditions, and better understanding of the mechanisms behind our findings may help to define ways to achieve protection against MS by other means than alcohol consumption.

Our study was designed as a population-based case–control study and information on exposures was collected retrospectively. To minimize the risk of recall bias, we predominantly included cases who had received their diagnosis within the past year. Recall bias usually results in overreporting of past exposures among cases or underreporting among controls, causing overestimation of any association between exposure and outcome. If our results would be explained by recall bias, it means that cases have systematically understated their previous alcohol consumption in relation to the statements among controls, or that controls have systematically overstated their alcohol consumption.

Another potential concern is that the recruitment of cases and controls may have introduced selection bias. However, the Swedish health care system provides free of charge access to medical services for all Swedish citizens, and we believe almost all cases of MS are referred to neurology units, and it seems unlikely that unidentified cases would cause a substantial bias in our calculations. Selection bias among controls is also likely to be modest since controls were selected from the population and the prevalence of smoking and alcohol consumption among controls, as well as their drinking patterns, was in line with those of the general population^38^. If selection bias had occurred among controls, it would probably be positively correlated with alcohol consumption, i.e. the response rate would probably be lower among those who drink large amounts of alcohol and consequently, our observed ORs would be biased towards the null value (OR = 1).

Reverse causation is another concern, i.e. that the disease has influenced the alcohol consumption among MS cases. Cases and controls were classified according to their alcohol habits at the time of study inclusion, which occurred after a median of 1.0 year after the disease onset. We performed a sensitivity analysis restricted to subjects with MS onset within five years prior to study inclusion who reported no change in alcohol consumption habits during the last five years, and our findings remained similar and statistically significant.

It can be argued that cases may experience decreased alcohol tolerance and therefore reduce their alcohol consumption before the onset of MS due to preexisting negative influences on the central nervous system (CNS) by the MS process. However, similar negative associations have been observed between alcohol consumption and the risk of other autoimmune disorders that do not directly affect the CNS^39–41^. Furthermore, in a recent Danish case–control study, alcohol consumption in adolescence was associated with lower risk of subsequent MS^19^. Although reverse causation is less likely to explain our findings, we cannot completely rule out this possibility.

We had the possibility to adjust for a large number of potential confounding factors, such as education, sun exposure habits and EBNA-1 antibody levels, and even if we cannot completely rule out a degree of bias in the estimated associations due to factors not considered in our study or due to residual confounding, we consider it unlikely that our findings would be affected by bias due to confounding to a large extent.

In conclusion, non-drinkers have an increased risk of developing MS compared to drinkers, and non-drinking interacts with DRB1*15:01 and smoking to increase the risk of the disease. Alcohol consumption has detrimental effects on other disease conditions, and better understanding of the mechanisms behind our findings may help to define ways to achieve protection against MS by other means than alcohol consumption.

## Supplementary Information

Supplementary Information.

## Data Availability

Anonymized data will be shared by request from any qualified investigator that wants to analyze questions that are related to the published article.

## Code availability

Code will be shared by request from any qualified investigator.
