# Supplementary material for: The increased risk of multiple sclerosis associated with HLA-DRB1*15:01 and smoking is modified by alcohol consumption
Source: Sci Rep. 2021 Oct 27;11:21237. doi: 10.1038/s41598-021-00578-y (PMC8551162; doi:10.1038/s41598-021-00578-y)
Supplement: Supplementary file 1 — Supplementary Information. [file 41598_2021_578_MOESM1_ESM.doc]

**The increased risk of multiple sclerosis associated with HLA-DRB1*15:01 and smoking is modified by alcohol consumption.**

**eTable 1**. OR with 95% CI of developing MS for subjects with different A*02:01 status and alcohol consumption habits. Attributable proportion due to interaction between absence of A*02:01 and non-drinking.

| A*02:01 | Alcohol | ca/co^1^ | OR (95% CI)^2^ | AP (95% CI) |
| --- | --- | --- | --- | --- |
| - | + | 599/1160 | 1.0 (reference) |  |
| - | - | 253/403 | 1.3 (1.0-1.5) |  |
| + | + | 833/943 | 1.7 (1.5-2.0) |  |
| + | - | 374/381 | 2.0 (1.6-2.3) | 0.005 (-0.2-0.2) |

^1^number of exposed cases and controls; ^2^adjusted for age, gender, residential area, smoking, and DRB1*15:01.

**eTable 2**. OR with 95% CI of developing MS for subjects with different alcohol consumption habits and DRB1*15:01, A*02:01, and smoking status. Attributable proportion due to interaction between non-drinking and DRB1*15:01, absence of A*02:01 and smoking, respectively.

| Pack years of smoking | Alcohol | ca/co^1^ | OR (95% CI)^2^ | AP (95% CI) |
| --- | --- | --- | --- | --- |
| 0 | + | 630/1076 | 1.0 (reference) |  |
| 0 | - | 336/525 | 1.2 (1.0-1.4) |  |
| <10 | + | 83/113 | 1.4 (1.2-1.6) |  |
| <10 | - | 36/16 | 1.6 (1.2-2.0) | 0.02 (-0.3-0.3) |
| Pack years of smoking | Alcohol | ca/co^1^ | OR (95% CI)^3^ | AP (95% CI) |
| 0 | + | 630/1076 | 1.0 (reference) |  |
| 0 | - | 336/525 | 1.2 (1.0-1.4) |  |
| 10-20 | + | 158/199 | 1.5 (1.2-2.0) |  |
| 10-20 | - | 83/48 | 3.5 (2.4-5.2) | 0.5 (0.5-0.7) |
| Pack years of smoking | Alcohol | ca/co^1^ | OR (95% CI)^4^ | AP (95% CI) |
| 0 | + | 630/1076 | 1.0 (reference) |  |
| 0 | - | 336/525 | 1.2 (1.0-1.4) |  |
| >20 | + | 561/715 | 1.4 (1.0-2.0) |  |
| >20 | - | 172/195 | 4.1 (2.2-7.7) | 0.6 (0.3-0.9) |

^1^number of exposed cases and controls; ^2^adjusted for age, gender, residential area, and DRB1*15:01 status.

**eTable 3**. OR with 95% CI of developing MS for subjects with different alcohol consumption habits and DRB1*15:01, A*02:01, and smoking status^1^. Attributable proportion due to interaction between non-drinking and DRB1*15:01, absence of A*02:01 and smoking, respectively.

| DRB1*15:01 | Alcohol | ca/co^2^ | OR (95% CI)^3^ | AP (95% CI) |
| --- | --- | --- | --- | --- |
| - | + | 459/991 | 1.0 (reference) |  |
| - | - | 205/419 | 1.2 (0.9-1.4) |  |
| + | + | 531/412 | 2.9(2.4-3.4) |  |
| + | - | 263/144 | 4.3 ( 3.4-5.4) | 0.3 (0.1-0.5) |
| A*02:01 | Alcohol | ca/co^2^ | OR (95% CI)^4^ | AP (95% CI) |
| - | + | 415/780 | 1.0 (reference) |  |
| - | - | 200/282 | 1.4 (1.1-1.7) |  |
| + | + | 575/623 | 1.8 (1.5-2.2) |  |
| + | - | 268/281 | 2.1 (1.7-2.6) | -0.07 (-0.3-0.2) |
| Smoking | Alcohol | ca/co^1^ | OR (95% CI)^5^ | AP (95% CI) |
| - | + | 451/724 | 1.0 (reference) |  |
| - | - | 254/372 | 1.2 (1.0-1.4) |  |
| + | + | 539/679 | 1.3 (1.1-1.6) |  |
| + | - | 214/191 | 1.9 (1.5-2.4) | 0.2 (0.01-0.4) |

^1^Analyses restricted to subjects with an index year within five years prior to inclusion in the study, who reported that they had not changed their alcohol consumption habits during the past five years.^2^number of exposed cases and controls; ^3^adjusted for age, gender, residential area, and smoking; ^4^adjusted for age, gender, residential area, smoking, and DRB1*15:01 status; ^5^adjusted for age, gender, residential area, and DRB1*15:01 status.

**eTable 4**. OR with 95% CI of developing MS for subjects with different alcohol consumption habits and DRB1*15:01. Attributable proportion due to interaction between low alcohol consumption and DRB1*15:01.

| DRB1*15:01 | Alcohol | ca/co^1^ | OR (95% CI)^2^ | AP (95% CI) |
| --- | --- | --- | --- | --- |
| - | + | 65/200 | 1.0 (reference) |  |
| - | - | 420/861 | 0.9 (1.4-2.7) |  |
| + | + | 82/78 | 1.9 (0.8-1.0) |  |
| + | - | 489/360 | 2.5 (2.1-3.0) | 0.3 (0.009-0.5) |

^1^number of exposed cases and controls; ^2^adjusted for age, gender, residential area, and smoking.

**eTable 5**. OR with 95% CI of developing MS for subjects with different alcohol consumption habits and smoking. Attributable proportion due to interaction between low alcohol consumption and smoking.

| Smoking | Alcohol | ca/co^1^ | OR (95% CI)^2^ | AP (95% CI) |
| --- | --- | --- | --- | --- |
| - | + | 40/100 | 1.0 (reference) |  |
| - | - | 454/702 | 1.1 (0.9-1.3) |  |
| + | + | 107/178 | 1.1 (0.8-1.4) |  |
| + | - | 455/519 | 1.5 (1.3-1.8) | 0.3 (0.03-0.5) |

^1^number of exposed cases and controls; ^2^adjusted for age, gender, residential area, and DRB1*15:01.
